# Temporal summation does not predict the acupuncture response in patients with chronic non-specific low back pain

**DOI:** 10.3389/fneur.2024.1335356

**Published:** 2024-08-23

**Authors:** Petra Baeumler, Margherita Schäfer, Luise Möhring, Dominik Irnich

**Affiliations:** Multidisciplinary Pain Center, Department of Anaesthesiology, LMU University Hospital, LMU Munich, Munich, Germany

**Keywords:** quantitative sensory testing, responder, central sensitization, wind-up ratio, cohort study, predictor, chronic pain

## Abstract

**Introduction:**

Previously, we had observed that immediate pain reduction after one acupuncture treatment was associated with high temporal summation of pain (TS) at a pain free control site and younger age in a mixed population of chronic pain patients. The aim of the present study was to verify these results in chronic non-specific low back pain (LBP) and to collect pilot data on the association between TS and the response to an acupuncture series.

**Methods:**

TS at a pain free control site (back of dominant hand) and at the pain site was quantified by the pin-prick induced wind-up ratio (WUR) in 60 LBP patients aged 50 years or younger. Response to one acupuncture treatment was assessed by change in pain intensity and pressure pain threshold (PPT) at the pain site. The primary hypothesis was that a high TS (WUR > 2.5) would be associated with a clinically relevant reduction in pain intensity of at least 30%. In study part two, 26 patients received nine additional treatments. Response to the acupuncture series was assessed by the pain intensity during the last week, the PPT and the Hannover functional ability questionnaire (FFbH-R).

**Results:**

An immediate reduction in pain intensity of at least 30% was frequent irrespective of TS at the control site (low vs. high TS 58% vs. 72%, *p* = 0.266). High TS at the pain site was also not significantly associated with a clinically relevant immediate reduction in pain intensity (low vs. high TS 46% vs. 73%, *p* = 0.064). The PPT was not changed after one acupuncture treatment. Study part two did not reveal a consistent association between TS at the control site and any of the outcome measures but also a trend toward a higher chance for a clinically relevant response along with low TS at the pain site.

**Conclusion:**

Our results do not suggest an important role of TS for predicting a clinically important acupuncture effect or the response to a series of 10 acupuncture treatments in patients with chronic non-specific LBP. Overall high response rates imply that acupuncture is a suitable treatment option for LBP patients irrespective of their TS.

## 1 Introduction

Acupuncture is used worldwide (1), with growing popularity (2–4). High quality randomized controlled trials support its effectiveness in the treatment of chronic pain (5). However, as for pharmacological pain management, just around half of chronic pain patients treated with acupuncture experience a clinically relevant improvement (6). In order to minimize adverse outcomes through ineffective pain treatment, the identification of patient characteristics that predict the effectiveness of acupuncture appears crucial. In the past, sociodemographic characteristics, disease severity and expectations of treatment outcomes have been studied the most, but revealed no substantial predictive value (7–11). Thus, the question arises whether response to acupuncture can be predicted on the basis of neurophysiological considerations. The analgesic effects of acupuncture involve local, segmental and central mechanisms (12–14). When it comes to treating chronic pain, the potential of acupuncture to modulate central sensitization processes seems to play an eminent role (15, 16). Consequently, we hypothesized that chronic pain patients with particularly pronounced signs of central sensitization might profit the most from acupuncture.

Elevated temporal summation of pain (TS) is a characteristic sensory sign in various chronic pain conditions that indicates facilitation of the ascending pain pathway (17–20). TS describes the increase of the perceived pain intensity during repetitive application of uniform pain stimuli. This usual physiological phenomenon arises from rapid facilitation of spinal synaptic transmission (also termed wind-up), and permanent augmentation of synaptic strength between nociceptive afferents and spinal projection neurons in chronic pain states can result in elevated TS (21). Various types of noxious stimuli can be used to elicit TS. In their validated and standardized protocol for quantitative sensory testing the German Research Network on Neuropathic Pain implemented the so called wind-up ratio (WUR) – the ratio of the pain intensity elicited by ten uniform pin-prick stimuli over the pain intensity elicited by one pin-prick stimulus (22).

Indeed, one of our previous studies showed a positive association between the pin-prick induced TS at a pain free control site and the immediate analgesic response to acupuncture in a mixed patient population suffering from different chronic pain conditions (23). Furthermore, we observed an independent impact of age. Conversely, results of this study did not support a robust relationship between the immediate response to acupuncture and the TS at the most painful body site.

The aim of the present study was to verify the association between high TS at a pain free control site and the immediate response to acupuncture in a patient population with a specific pain condition, namely chronic non-specific low back pain (LBP). A secondary aim of this study was to collect pilot data about potential associations between the TS at a pain free control site and the pain site at the lower back with the response to a series of ten acupuncture treatments.

LBP was chosen for the following reasons: First, LBP is one of the most frequent chronic pain conditions (24–27), and a large part of LBP is non-specific with central sensitization processes constituting an important component of chronic LBP (28, 29). Second, acupuncture is recommended for its treatment (30, 31) while not negating limitations of the existing evidence (32). Third, as for other chronic pain conditions responder rates among chronic LBP patients after acupuncture have been estimated to be around 50% (33).

In addition to pain intensity, the pressure pain threshold (PPT) at the lower back and LBP related functional disability were chosen as additional outcome measures in the present study to further objectify our findings. The PPT at the lower back of LBP patients has been shown to be reduced in comparison to healthy controls (34, 35). Furthermore, the PPT has been shown to be reliably increased by acupuncture in healthy subjects and pain patients (36, 37); particularly in LBP patients either through dry needling of myofascial trigger points or through needling at traditional acupuncture points (38–42).

## 2 Methods

### 2.1 Study design

Aim of this prospective cohort study was to evaluate whether the previously identified association between high TS and the immediate pain relief through acupuncture in a mixed population of patients suffering from different chronic pain conditions (23) can be confirmed in a more narrowly defined population of patients with chronic non-specific LBP. Due to the impact of age on this association observed in the previous study, patients age in this trial was restricted to 50 years or younger. The acupuncture treatments followed a semi-standardized regimen used in the large German acupuncture trials on LBP (43). The immediate acupuncture effect was evaluated by assessing the current pain intensity and the PPT at the pain site before and directly after treatment. An immediate reduction of the pain intensity of at least 30% was defined as the primary outcome measure. Before acupuncture treatment, an independent examiner assessed TS as the pin-prick induced WUR at the pain site and at the dorsum of the dominant hand which served as a control site. Examiners assessing the outcome measures were blinded for the patients’ TS.

Additionally, the second part of this study was designed to collect data about whether TS might also predict the treatment effect of a whole acupuncture series. Therefore, the study protocol foresaw that two subgroups of patients with high TS (WUR ≥ 3.5) and low TS (WUR ≤ 2.0) at the control site would receive nine additional acupuncture treatments (ten treatments in total over four weeks with a maximum of three treatments per week). Against the protocol, four patients with a WUR between 2.6 and 3.0 were accidently also included in this second study part. The treatment effect of the whole acupuncture series was evaluated by assessing the pain intensity and back pain-related disability (Hannover functional ability questionnaire, FFbHR) during the last week before inclusion and in the week after the last acupuncture treatment. Additionally, the PPT was again assessed at the last study visit 1 week after treatment termination. As in study part one, examiners performing the outcome assessments were blinded against the patients’ TS.

### 2.2 Patients

General inclusion criteria were age between 18 and 50 years, good command of the German language and written informed consent. The restriction to adult patients aged 50 years or younger was based on our previous observation, that the association between TS and the acupuncture response was most pronounced in this age group (23).

Specific inclusion criteria were the diagnosis of chronic non-specific LBP as defined by the German National Disease Management Guideline [*Nationale Versorgungsleitline nicht-spezifischer Kreuzschmerz* (44)] with the main pain site being located in the segments L1 to L5, a pain duration of more than three months and an average pain intensity during the week prior to inclusion of over 40 on a 100 mm visual analog scale (VAS). Exclusion criteria were specific LBP, acute necessity of further diagnostic or therapeutic procedures, previous back/spine surgery, malignant rheumatoid or chronic inflammatory diseases, severe psychiatric disorders, previous major depression, pregnancy, regular intake of opioids, antidepressants or anticonvulsants, acute or chronic complaints at the hands and acupuncture within the last 6 months.

Patients were recruited through information sheets displayed in pharmacies and primary care facilities (private medical and physiotherapist practices), posters displayed at the Munich railway station and in employee facilities of the University Hospital LMU Munich. In addition, notifications about the study were sent twice to an LMU e-mail distribution list of voluntary recipients. Interested patients were first screened per telephone. Remaining patients were invited to the study center. After written informed consent was obtained, patients were subjected to an initial medical examination during which the diagnosis of specific or non-specific LBP was established through evaluation of red flags, and the necessity of immediate further diagnostic or therapeutic procedures was determined.

### 2.3 Intervention

Acupuncture treatments were performed by experienced acupuncturists, who were medical doctors with at least 300 hours of acupuncture training and at least ten years of acupuncture practice. Treatments followed a semi-standardized protocol corresponding to the acupuncture regimen applied in the large health insurance sponsored German acupuncture trials (43). This regimen combines obligatory and facultative points. Out of 27 local/segmental points (BL 20 to BL 34, BL50 to BL 54, GB 30, GV 3, GV 4, GV 5, GV 6, EX-B 2, EX-B 9) at the lower back, at least four had to be chosen and needled bilaterally (except for governor vessel points). In case of pseudoradicular symptomatology two further local points were supposed to be selected. Furthermore, out of twelve distal points (SI 3, BL 40, BL 60, BL 62, KI 3, KI 7, GB 31, GB 34, GB 41, LR 3, GV 14, GV 20) at least two were to be selected and needled bilaterally; except for governor vessel (GV) points. Thus, depending on the presence of a pseudoradicular symptomatology the minimum number of obligatory needles was twelve or 16, respectively. Acupuncturists were free to choose further body or microsystem points, but the total number of needles was not supposed to exceed 20. Acupuncture points were selected according to a thorough TCM anamneses performed before the first treatment and adjusted according to the patients’ reactions during the subsequent treatments. Acupuncturists were free to choose needle size and to stimulate the needles either manually by rotation or up and down movement or electrically with Han-frequency. Needle stimulation was adjusted to be intense but not painful. At each needle insertion elicitation of deqi was intended but not forced. Resting time with needles in place was 25 minutes at minimum. Acupuncture treatment was initiated at the day of inclusion in all but one case who received the first acupuncture treatment on the day subsequent to inclusion. In case of participation in the second study part patients received ten acupuncture treatments in total within four weeks with a maximum of three treatments per week. Adverse events were documented by the acupuncturists and examiners. Patients were queried before each treatment about eventual adverse events related to the previous acupuncture and after each treatment about adverse events of the recent acupuncture.

### 2.4 Outcome measures and predictor variables

#### 2.4.1 Pain intensity

The primary outcome was the occurrence of a reduction in current pain intensity of at least 30% immediately after one acupuncture treatment which was shown to represent a clinically meaningful pain relief (45). According to recommendations of the US Initiative on Methods, Measurement, and Pain Assessment in Clinical Trials [IMMPACT (46, 47)] the occurrence of a reduction in current pain intensity of at least 50% was also reported. Pain intensity was evaluated by means of a 100 mm VAS (0 = no pain, 100 = maximum imaginable pain). The current pain intensity was determined directly before and after the first acupuncture treatment always prior to the assessment of the PPT and the TS.

The average pain intensity during the week prior to inclusion was determined in the course of the verification of in- and exclusion criteria on the day of inclusion. In case of participation in the second study part the average pain intensity during the week after treatment termination was assessed on a separate study visit. For the reduction in the average pain intensity during the last week, it was also documented whether it attained at least 30% or 50%, respectively.

Percent changes in pain intensity were calculated as follows with negative percent changes indicating an improvement.


$$\%\mathrm{\Delta VAS}=\frac{\left(VAS_{\mathrm{post}}-VAS_{\mathrm{baseline}}\right)}{VAS_{\mathrm{baseline}}}*100$$

#### 2.4.2 Pressure pain threshold

The pressure pain threshold (PPT) was determined at the most painful segment at the lower back (two finger breadths lateral to the spinous process). PPT assessments adhered to the protocol of quantitative sensory testing established by the German Research Network on Neuropathic Pain (22). Pressure was applied by a Fisher-algometer (FDK20, 2–10 kg/cm2, Wagner Instruments, Greenwich, CT, United States). The force was increased by 0.5 kg/cm^2^ per second until the patients indicated the onset of pressure pain. The arithmetic mean of three measurements were calculated to represent the final PPT score. Time points of PPT assessments were before and after the first acupuncture treatment as well as one week after the tenth acupuncture treatment in patients taking part in the second study part. In accordance with the definition of a clinically relevant pain relief, we also documented whether the PPT had increased by at least 30% at the two time points in comparison to baseline. A 30% elevation of the PPT appears clinically relevant according to following consideration: The PPT at paravertebral measure sites at the lower back in LBP patients has been estimated to lie around 3 kg/cm^2^ (35). Thus, a 30% elevation would correspond to 1.05 kg/cm^2^. Changes of this magnitude were observed after dry needling at similar measure sites in LBP patients (42) as well as in one of our own previous trials after segmental needling at the leg in healthy volunteers (36).

Percent changes in the PPT were calculated as follows: $\%\Delta \;PPT=\frac{\left(PPT_{\mathrm{post}}-PPT_{\mathrm{baseline}}\right)}{PPT_{\mathrm{baseline}}}*100$ .

As higher thresholds indicate that higher pressure is required to elicit pain, positive percent changes indicate an improvement.

#### 2.4.3 Hannover functional ability questionnaire

The FFbH-R is a twelve-item questionnaire quantifying the functional ability in activities of daily living among patients with LBP with satisfactory internal consistency, high test–retest reliability and sensitivity to change (48, 49). Items are scored on a three-point scale (2 = performance of task without difficulties; 1 = performance of task with difficulties; 0 = performance of task only with help from others). The final FFbH-R score is calculated according to Kohlmann and Raspe (48) by rescaling the mean of all valid items to a score between 0% (worst functional ability) and 100% (best functional ability). Patients filled out the FFbHR before the first acupuncture treatment and in study part two at the follow-up visit one week after termination of the tenth acupuncture treatment.

As for the other outcomes, cases were categorized in those with a 30% or larger improvement and those with less than 30% improvement. Percent changes in the FFbHR score were calculated as follows: $\%\Delta \;\mathrm{FFbHR}=\frac{\left({\mathrm{FFbHR}}_{\mathrm{post}}-{\mathrm{FFbHR}}_{\mathrm{baseline}}\right)}{{\mathrm{FFbHR}}_{\mathrm{baseline}}}*100$ .

As higher scores indicate better functioning, positive percent changes indicate an improvement.

#### 2.4.4 Temporal summation

Temporal summation (TS) was quantified by the pin-prick induced wind-up ratio (WUR) according to the protocol of quantitative sensory testing established by the German Research Network on Neuropathic Pain (22). The WUR is calculated as the ratio of the pain rating evoked by ten pin-prick stimuli over the pain rating evoked by one pin-prick stimulus. The pin-prick evoked pain is rated on a verbal rating scale (0–100). We calculated the mean of three WUR assessments conducted at an interval of five minutes each at the control site (dorsum of the dominant hand) and at the pain site (two finger breadths lateral to the spinous process). To avoid an impact of the TS assessments on the subsequent PPT assessments at the pain site, patients were asked to wait and relax for 15 min between the two tests. Pin-pricks (MRC Systems GmbH-Medizintechnische Systeme, Heidelberg, Germany) of either 128, 256, or 512 mN were used according to the patient’s sensitivity in the respective measurement area. Floor and ceiling effects were thereby omitted.

#### 2.4.5 Patient characteristics

On the day of inclusion the patients’ age, sex, pain duration and current medication were documented.

### 2.5 Biometry

#### 2.5.1 Sample size calculation

The sample size estimation was based on the results of our previous study among patients below the age of 53 years (23). We anticipated a percentage of patients with high TS of 40%, an average responder rate of 44% and an odds ratio (OR) of 4.5 for the association between clinically relevant acupuncture response and high TS. A sample size calculation based on these parameters, a power of 80% and an alpha error of 5% resulted in 56 required patients which we rounded to 60 in order to account for drop-outs.

#### 2.5.2 Data analysis

Due to the skewed distributions of some continuous variables, descriptive statistics are presented in this manuscript as medians and interquartile ranges. Categorical variables are presented as absolute and relative frequencies. Differences between outcomes at baseline and at follow-up time points were evaluated by the Wilcoxon rank sum test.

As described above, outcomes were dichotomized at a cut-off of ≥30% improvement reflecting a clinically relevant change. The percent change of the current pain intensity was also dichotomized at a cut-off of ≥50%. According to the primary study hypothesis based on our previous study, TS at the control and the pain site was categorized into high WUR (>2.5) and low WUR (≤2.5). Logistic regression models were used to assess whether high TS at the control or pain site predicted the likelihood to experience a clinically relevant immediate response to one acupuncture treatment as indicated by the dichotomized outcomes. In order to assess potential confounding the following patient characteristics were included as covariates: age and sex, use of analgesics, pain duration, baseline pain intensity (current and during last week before inclusion), baseline PPT and baseline FFbHR scores. Separate models were used in order to avoid multicollinearity. Adjusted analyses were omitted for mutually exclusive categories.

The Mann–Whitney-U test was used to compare percentage changes in outcomes between patients with high and low TS. Comparisons of baseline values and patient characteristics between patients with and without a clinically relevant pain relief after one or ten acupuncture treatments, respectively, were conducted by Mann–Whitney-U test for continuous variables and by Fisher’s test for dichotomous variables.

The relationship between percent changes in outcome variables and TS at the control and pain site in their uncategorized, continuous form were explored graphically in scatter plots and by generalized linear models implementing maximum likelihood estimation and an identity link function. The WUR and the PPT approached a log-normal distribution and were thus log-transformed before inclusion in GLM-models.

The pilot data collected in the second study part on the relationship between the responses to a series of ten acupuncture treatments and TS at the control and the pain site were only descriptively analyzed. These data were used to calculate the sample sizes that would be required in eventual confirmatory trials on the predictive value of TS for the response to acupuncture.

### 2.6 Ethics

The present investigation was approved by the Ethics Committee of the Medical Faculty of the Ludwig-Maximilians University (LMU), Munich, Germany and was performed in accordance with the declaration of Helsinki (50). Data management and storage adhered to the German data-protection act. Study data were pseudonymized and are kept separate from personal information in the research facilities of the Multidisciplinary Pain Center, Department of Anaesthesiology, LMU University Hospital, LMU Munich, Germany for ten years. All participating patients gave voluntary written informed consent and were free to withdraw from the study at any point. Patients were remunerated with 40 Euro for completion of study part one and with 85 Euro for completion of study part two. Patients who visited the study center for the screening visit, but did not meet in- or exclusion criteria were compensated with ten Euro.

## 3 Results

### 3.1 Patient characteristics

**Figure 1:**
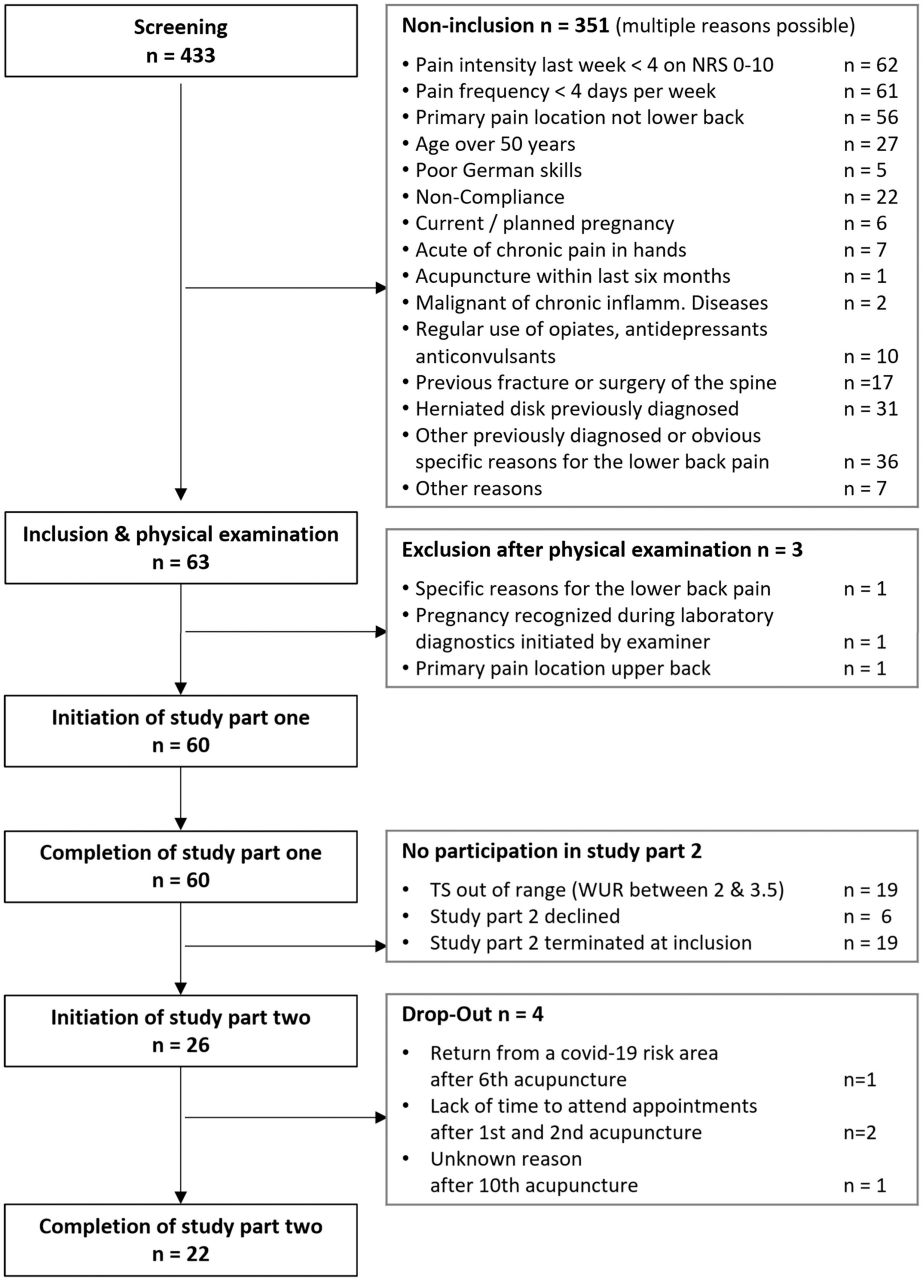
Study flow diagram.

In total 433 patients were screened. Reasons for non-inclusion are displayed in Figure 1. Sixty-three patients provided written informed consent to participate in the study. Three of those were excluded again after the physical examination. Thus, 60 patients were finally subjected to study part one which was completed on the day of inclusion by 59 patients and on the subsequent day by one patient. Twenty-six patients were allocated to study part two, but four dropped out before the final visit, resulting in 22 complete data sets (Figure 1).

The majority of patients were female (75%), and the median age was 26.0 years (IQR [21.0; 38.8]). Pain duration varied between four months and 32 years with a median of 45.5 months [14.3; 91.5]. Main pain was most prevalent in the segments L4 and L5 (78%) and at the right body side (69%). About a third of patients used analgesics on demand. Among those 21 patients, 17 used oral non-steroidal anti-inflammatory drugs (NSAID), three paracetamol, two metamizole and one a topical NSAID. In addition, five patients (8%) were on vitamin D or vitamin B12 supplementation and eight (13%) on thyroxine supplementation. In all but one patient, the dominant hand was the right hand that served as the control site for the determination of the TS. 1.5 cun, used to define the measure site at the back, varied between three and four centimeters with a median of 3.4 cm [3.2; 3.7]. In around half of the cases, a pin-prick of 256 mN strength was used for TS determination, in one third a pin-prick of 512 mN. Median TS as evaluated by the WUR varied between 1.1 and 13.8 at the control site and between 1.0 and 7.5 at the pain site with similar medians (1.9 [1.5; 3.3] and 1.6 [1.4; 2.5]). 40% of patients exhibited a WUR of over 2.5 at the control site and 25% showed a WUR over 2.5 at the pain site. Characteristics of patients participating in study part two resembled the total population (Table 1).

**Table 1:** Patient characteristics.

		Study part 1	Study part 2
		*n* = 60	*n* = 22
Age (years), median [IQR]		26.0 [21.0; 38.8]	30.5 [22.8; 43.3]
Female, *n* (%)		45 (75)	18 (82)
Pain duration (months), median [IQR]		45.5 [14.3; 91.5]	48.5 [14.5; 96.3]
Segment of main pain, *n* (%)	L1	6 (10)	2 (9)
	L2	3 (5)	0
	L3	4 (7)	2 (9)
	L4	23 (38)	8 (36)
	L5	24 (40)	10 (45)
Body side of main pain, *n* (%)	Left	18 (31)	7 (32)
	Right	40 (69)	15 (68)
Analgesic medication, *n* (%)		21 (35)	5 (23)
Vitamin D or B12, *n* (%)		5 (8)	2 (9)
Thyroxine, *n* (%)		8 (13)	5 (23)
Dominant hand, *n* (%)	Left	1 (2)	0
	Right	59 (98)	22 (100)
2 finger breadths (cm), median [IQR]		3.4 [3.2; 3.7]	3.4 [3.2; 3.7]
Pin-prick for TS assessment control site, *n* (%)	128 mN	8 (13)	2 (9)
	256 mN	32 (53)	10 (45)
	512 mN	20 (33)	10 (45)
Pin-prick for TS assessment pain site, *n* (%)	128 mN	11 (18)	4 (18)
	256 mN	31 (52)	12 (55)
	512 mN	18 (30)	6 (27)
TS as WUR (NRS-ratio), median [IQR]	Control site	1.9 [1.5; 3.3]	1.9 [1.6; 3.5]
	Pain site	1.6 [1.4; 2.5]	1.6 [1.4; 2.6]
High TS (WUR > 2.5), *n* (%)	Control site	24 (40)	10 (45)
	Pain site	15 (25)	5 (23)

IQR, interquartile range; cm, centimeters; mN, millinewton; TS, pin-prick induced temporal summation of pain as evaluated by the wind-up ratio (WUR) with WUR calculated as pain intensity on the numeric rating scale (NRS 0–100) evoked by 10 stimuli divided by pain intensity evoked by one stimulus (high TS (WUR > 2.5) indicates central sensitization).

### 3.2 Treatment characteristics

Of the 273 acupuncture treatments conducted, 264 (97%) were in accordance with the semi-standardized protocol. At the discretion of the acupuncturist, the number of needles was reduced in six treatments and increased in three treatments. The number of needles per acupuncture session varied between 13 and 22. The median number of needles was 19 (IQR [18; 20], min-max [13–22]). Points most commonly used in the first treatment resembled those most commonly used in the subsequent treatments (Supplementary Table 1). Most common points out of the set for obligatory points were BL 23, BL 25, BL 40, GB 34, and KID 3. These were used in over 50% of the initial acupuncture treatments; BL 23 and BL25 even in 90% of treatments. Obligatory points needled in at least 30% to 50% of all initial treatments were BL 27, LR 3, GV 4, BL 26, and BL 24. Most commonly treated facultative points (30% to 63% of treatments) were microsystem points at the ear, namely the ear zone for the lumbar spine as well as the ear points Shenmen and Jerome. Most commonly used needles had a diameter of 0.2 mm (63% of all needles), followed by 0.3 mm diameter needles (29%) and 0.16 mm diameter needles (8%). In single cases also smaller needles (diameter 0.15 or 0.12 mm) were used. In all treatments acupuncturists decided not to use electrical needle stimulation.

Twenty six patients reported a total of 42 adverse events during or following 35 treatments. All adverse events resolved latest within the next days. None of the patients discontinued treatment or withdrew from the study because of adverse events. Most frequently reported events were small haematoma (*n* = 6) and bleeding (*n* = 4) at the needling site, pain after needle insertion (*n* = 5) or after needle withdrawal (*n* = 8), transient paraesthesia after needle withdrawal (*n* = 6) and vertigo directly after treatment (*n* = 5). Adverse events that occurred once during or following one of the acupuncture treatments were an emotional reaction with crying, a cramp in the plantar part of the foot, muscular tension in the area of the sacroiliac joint, flatulence with diarrhea, nausea, transient aggravation of symptoms and vertigo on the first post-treatment day. None of the adverse events were serious or required treatment.

### 3.3 Change in outcome measures over time

Change in outcome measures over time are depicted in Table 2. Current pain was significantly reduced immediately after the first acupuncture treatment, as was the average pain intensity during the last week evaluated one week after the tenth treatment. Two thirds of patients (67%, *n* = 40) experienced an immediate pain reduction of 30% or more and half of the patients (*n* = 30) an immediate pain reduction of 50% or more after the first acupuncture treatment. Among patients in study part two, a reduction in average pain during the last week of at least 30% or 50% was reported by 82% (*n* = 18) and 68% (*n* = 15) of patients, respectively, one week after the tenth treatment.

**Table 2:** Change in outcome measures over time.

Time point	Current pain	Pain last week	PPT	FFbHR
	VAS (0–100)	VAS (0–100)	kg/cm^2^	(0–100)
	median [IQR]	median [IQR]	median [IQR]	Median [IQR]
**Study part 1 *n* = 60**				
Baseline (t0)	45.0 [33.0; 55.0]	58.5 [50.0; 62.8]	3.4 [2.3; 4.4]	83.3 [75.0; 91.7]
Post 1st acu (t1)	25.5 [9.3; 34.8]	-	3.3 [2.2; 4.8]	-
*p*-value	<0.001^*^	-	0.466	-
Crude change ∆t0–t1	−17.5 [−32.8; −5.5]	-	−0.2 [−0.5; 0.5]	-
Percent change %∆t0–t1	−47.2 [−75.3; −17.8]	-	−5.8 [−15.6; 14.3]	-
**Study part 2 *n* = 22**				
Baseline (t0)	44.5 [33.8; 55.5]	60.5 [49.8; 65.8]	3.5 [2.1; 4.3]	81.3 [72.9; 87.5]
1w after 10th acu (t2)	-	19.5 [11.5; 39.5]	4.0 [2.5; 5.7]	89.6 [83.3; 100.0]
*p*-value	-	< 0.001*	0.006^*^	0.014^*^
Crude change ∆t0–t2	-	−35.5 [−43.3; −22.3]	0.7 [−0.3; 1.8]	4.2 [−1.0; 20.8]
Percent change %∆ t0–t2	-	−65.9 [−80.2; −35.5]	17.9 [−11.9; 39.4]	4.7 [−1.1; 26.7]

Acu, acupuncture; VAS, visual analog scale; PPT, pressure pain threshold; FFbHR, Hannover functional ability questionnaire; IQR, interquartile range; ^*^Statistically significant change from baseline on an α-level of 5% as evaluated by the Wilcoxon rank sum test.

The PPT at the pain site remained unchanged immediately after the first acupuncture treatment, but was significantly elevated one week after the tenth treatment among patients participating in study part two. Only 13% (*n* = 8) of patients experienced an elevation of the PPT of at least 30% after the first acupuncture treatment. One week after the tenth acupuncture treatment, the PPT was elevated by at least 30% in 36% (*n* = 8) of the patients in study part two.

FFbHR scores significantly improved between baseline and one week after the tenth treatment. At baseline, 5% of the patients (*n* = 3) showed clinically relevant impairments in physical functioning (FFbHR < 60), 42% (*n* = 25) showed moderate impairments (FFbHR < 80/≥60) and 53% showed normal physical functioning (FFbHR ≥ 80) [categorization according to Kohlmann & Raspe (48)]. A similar distribution of FFbHR baseline scores was found in patients participating in study part two. One week after ten acupuncture sessions the proportion of patients with normal physical functioning had increased to 82, and 18% (*n* = 4) had experienced an increase of the FFbHR score of at least 30%.

### 3.4 Association between temporal summation and the immediate acupuncture response (study part one)

#### 3.4.1 Primary endpoint: association between temporal summation at the dorsum of the hand with a clinically relevant pain relief immediately after one acupuncture treatment

In contrast to the primary study hypothesis, immediate analgesic response to one acupuncture treatment was not positively associated with high TS at the dorsum of the hand (control site). The chance to experience a reduction of the current pain intensity of at least 30% or 50% did not differ significantly between patients with a high TS (WUR > 2.5) and low TS (WUR ≤ 2.5) at the control site (Table 3). A 30% reduction in current pain was experienced by 72% of patients with a low TS and by 58% of patients with a high TS at the control site (OR [95%-CI] 0.54 [0.18; 1.60], *p* = 0.266). The proportions of patients with a 50% reduction in current pain were 53% among patient with low TS and 46% among patients with high TS, respectively (OR [95%-CI] of 0.76 [0.27; 2.13], *p* = 0.598). Adjusted logistic regression analyses did not indicate any change in OR estimates neither through age, sex, pain duration, use of analgesics, baseline current pain, pain during the week before inclusion, baseline PPT nor through the baseline FFbHR score (Supplementary Table 2).

**Table 3:** Differences of response to first acupuncture between patients with a high and low temporal summation.

After 1st acupuncture		WUR ≤ 2.5	WUR > 2.5	OR [95%-CI]	*p*-value
		**TS at control site**		**Log regression**	
%∆ VAS	No	10	10	0.54 [0.18; 1.60]	0.266
≥30%	Yes	26	14		
%∆ VAS	No	17	13	0.76 [0.27; 2.13]	0.598
≥50%	Yes	19	11		
%∆ PPT	No	31	21	0.89 [0.19; 4.11]	0.877
≥30%	Yes	5	3		
		**TS at pain site**		**Log regression**	
%∆ VAS	No	12	8	0.32 [0.09; 1.07]	0.064
≥30%	Yes	33	7		
%∆ VAS	No	20	10	0.40 [0.12; 1.36]	0.142
≥50%	Yes	25	5		
%∆ PPT	No	37	15	Fisher’s test	0.182
≥30%	Yes	8	0		

TS, temporal summation as evaluated by the wind-up ratio (WUR with high TS (WUR > 2.5) indicating central sensitization); Log regression, logistic regression; OR, odds ratio; 95%-CI, 95% confidence interval; %∆ VAS, percent reduction in current pain intensity as evaluated by the visual analog scale; %∆ PPT, percent elevation in pressure pain threshold; More detailed model characteristics and covariate adjusted models are depicted in Supplementary Table 2.

**Figure 2:**
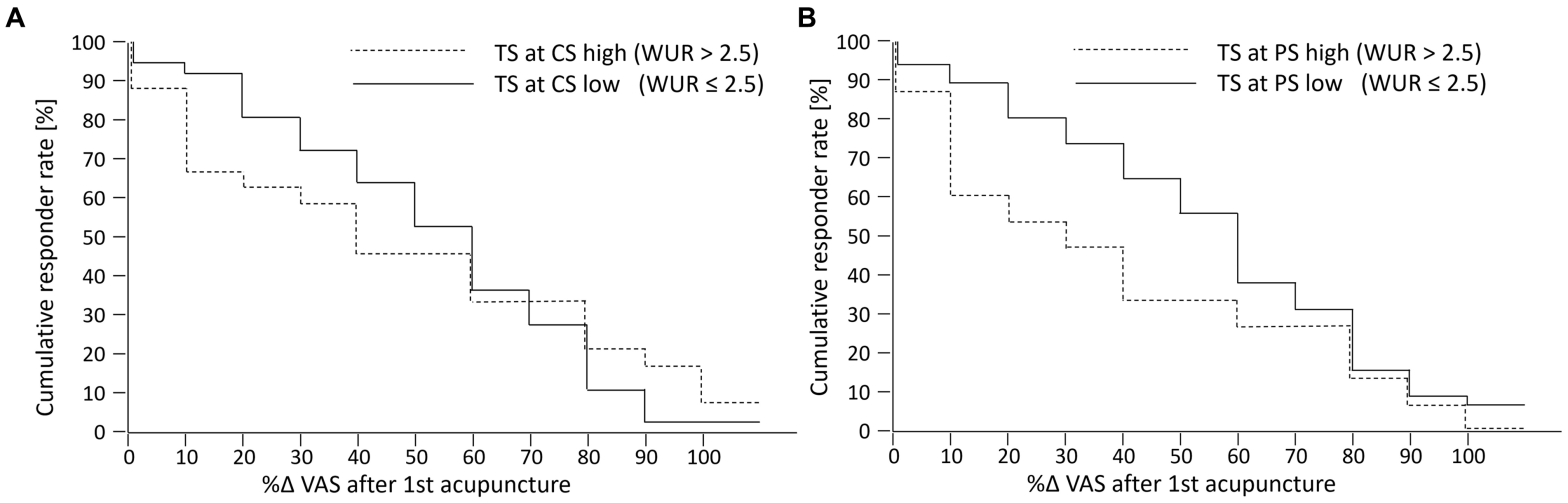
**(A, B)** Cumulative response function of percent change in current pain intensity after first acupuncture. TS, temporal summation as evaluated by the wind-up ratio (WUR with high TS (WUR > 2,5) indicating central sensitization); CS, control site at the dorsum of the dominant hand; PS, pain site at the lower back; %∆; VAS, percent change in current pain intensity as evaluated by the visual analog scale (0–100).

The cumulative response function illustrates that, irrespective of the responder definition, responder rates after one acupuncture treatment of patients with high and low TS at the control site did not differ importantly (Figure 2A). Likewise, the median percentage change in current pain intensity did not differ between patients with low and high TS at the control site (−50.0% [−74.2%; −23.6%] vs. −36.2% [−77.2%; −1.1%], MW-test *p* = 0.478).

#### 3.4.2 Secondary endpoints for associations between temporal summation and immediate effects of one acupuncture treatment

High TS at the pain site was also not significantly associated with a clinically relevant immediate reduction of current pain intensity after the first acupuncture treatment. There was only a non-significant trend toward a higher chance for a reduction of the current pain intensity of at least 30% in patients with a low TS at the pain site (WUR ≤ 2.5) than in patients with a high TS at the pain site (WUR > 2.5); 73% vs. 46% (Table 3). This trend also remained in the adjusted logistic regression analyses (Supplementary Table 2). The cumulative response function also illustrates that higher responder rates among patients with a low TS at the pain site occurred largely independent from the responder definition (Figure 2B). Accordingly, the median percentage change in current pain intensity tended to be larger in patients with a low TS than patients with high TS at the pain site (−50.9% [−75.6%; −25.1%] vs. −29.3% [−70.2%; 0.0%], MW-test *p* = 0.077).

The chance for an increase of the PPT of at least 30% did not differ between patients with a high and those with a low TS at the control site (13% vs. 14%, Table 3). Logistic regression with adjustment for covariates did also not indicate that TS at the control site was associated with the likelihood for an increase in the PPT of at least 30% (Supplementary Table 2). In contrast, none of the patients with a high TS at the pain site experienced an elevation of the PPT of at least 30%, while 18% of the patients with a low TS at the pain site showed such immediate response (Table 3). The median PPT percent change did neither differ between patients with low and high TS at the control site (−12.6% [−20.3%; 6.0%] vs. 0.6% [−11.9%; 25.0%], MW-test *p* = 0.083) nor between patients with low and high TS at the pain site (−7.8% [−16.9%; 13.5%] vs. −3.4% [−13.5; 20.5], MW-test *p* = 0.739).

**Figure 3:**
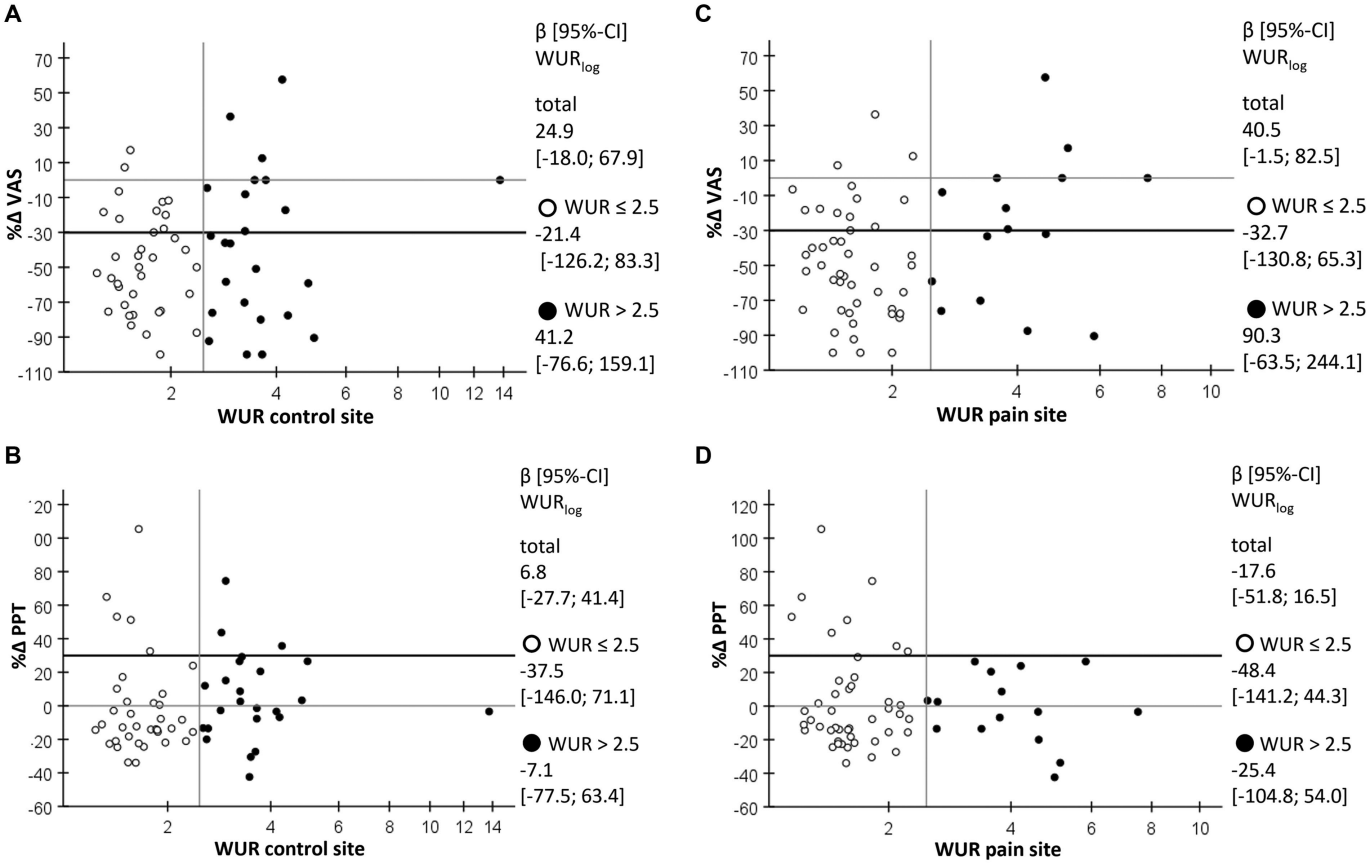
Scatterplot of percent change in outcomes after the first acupuncture against TS at the control and the pain site. **(A)** Percent change in current pain intensity (%∆ VAS) as evaluated by the visual analog scale against the wind-up ratio (WUR) as a measure for temporal summation of pain at the control site with high TS (WUR > 2.5) indicating central sensitization. **(B)** Percent change in pressure pain threshold (%∆ PPT) against the WUR at the control site. **(C)** Percent change in current pain intensity (%∆ VAS) as evaluated by the visual analog scale against the WUR at the pain site. **(D)** Percent change in pressure pain threshold (%∆ PPT) against the WUR at the pain site; empty circles, cases with WUR ≤ 2.5; filled circles, cases with WUR > 2.5; β [95%-CI] WURlog, regression coefficient of logarithmized WUR as estimated by generalized linear model.

In Figure 3 the log-transformed WUR at the control and the pain site are plotted against the percent change in current pain intensity and PPT, respectively. Regression analysis of the total sample and the two strata with high and low TS did not reveal any significant linear relationship. Again, there was a trend toward larger reductions in the current pain intensity along with lower TS at the pain site (β [95%-CI] WUR_log_ 40.5 [−1.5; 82.5], *p* = 0.059, Figure 3C).

Patient characteristics and baseline values of outcomes did not differ between patients with a reduction in pain intensity of at least 30% or 50% and those without such response after the first acupuncture treatment (Supplementary Table 3).

### 3.5 Association between temporal summation and the response to a series of acupuncture treatments (study part two)

The second study part foresaw the inclusion of patients with a substantially low TS (WUR ≤ 2.0) and high TS (WUR ≥ 3.5) at the control site. A protocol violation caused an additional inclusion of four patients with a TS between 2.6 and 3.0 at the control site. In the following, we describe both, the per protocol (PP)-data and all patient data obtained in study part two (intention to treat (ITT)-data). Descriptive statistics of both, the ITT- and the PP-data, indicate that, patients with a low TS at the pain site showed a reduction in the pain intensity within the last week of at least 30% or 50% as well as an increase in the PPT and the FFbHR score of at least 30% more frequently than those with a high TS. Association between TS at the control site and outcomes were less clear, but all OR were also below one (Table 4).

**Table 4:** Differences of response to ten acupuncture treatment between patients with a high and low temporal summation.

		Intention to treat analysis			Per protocol analysis		
		WUR ≤ 2	WUR > 2.5	OR [95%-CI]	WUR ≤ 2	WUR ≥ 3.5	OR [95%-CI]
		**TS at control site**			**TS at control site**		
%∆ VAS	No	2	2	0.80 [0.09; 7.00]	2	2	0.40 [0.04; 3.90]
≥30%	Yes	10	8		10	4	
%∆ VAS	No	3	4	0.50 [0.08; 3.08]	3	4	0.17 [0.02; 1.42]
≥50%	Yes	9	6		9	2	
%∆ PPT	No	7	7	0.60 [0.10; 3.54]	7	5	0.28 [0.02; 3.19]
≥30%	Yes	5	3		5	1	
%∆ FFbHR	No	9	9	0.33 [0.03; 3.84]	9	6	
≥30%	Yes	3	1		3	0	
		**TS at pain site**			**TS at pain site**		
%∆ VAS	No	2	2	0.20 [0.02; 2.03]	2	2	0.17 [0.01; 1.96]
≥ 30%	Yes	15	3		12	2	
%∆ VAS	No	4	3	0.21 [0.02; 1.69]	4	3	0.13 [0.01; 1.70]
≥50%	Yes	13	2		10	1	
%∆ PPT	No	10	4	0.36 [0.03; 3.92]	8	4	
≥30%	Yes	7	1		6	0	
%∆ FFbHR	No	13	5		11	4	
≥30%	Yes	4	0		3	0	

TS, temporal summation as evaluated by the wind-up ratio (WUR with high TS (WUR > 2.5) indicating central sensitization); OR, odds ratio; 95%-CI, 95% confidence interval; %∆ VAS, percent reduction in current pain intensity as evaluated by the visual analog scale; %∆ PPT, percent elevation in pressure pain threshold; %∆ FFbHR, percent elevation in the sum score of the Hannover Functional Ability Questionnaire.

Sample size calculations based on these pilot data (per protocol data, alpha level 0.05 and 80% power) resulted in 78 patients for the association between the likelihood of a pain reduction of at least 30% after ten acupuncture treatments with a low TS at the pain site at the lower back and in 240 patients for the association between the likelihood of a pain reduction of at least 30% after ten acupuncture treatments with a low TS at the dorsum of the hand.

**Figure 4:**
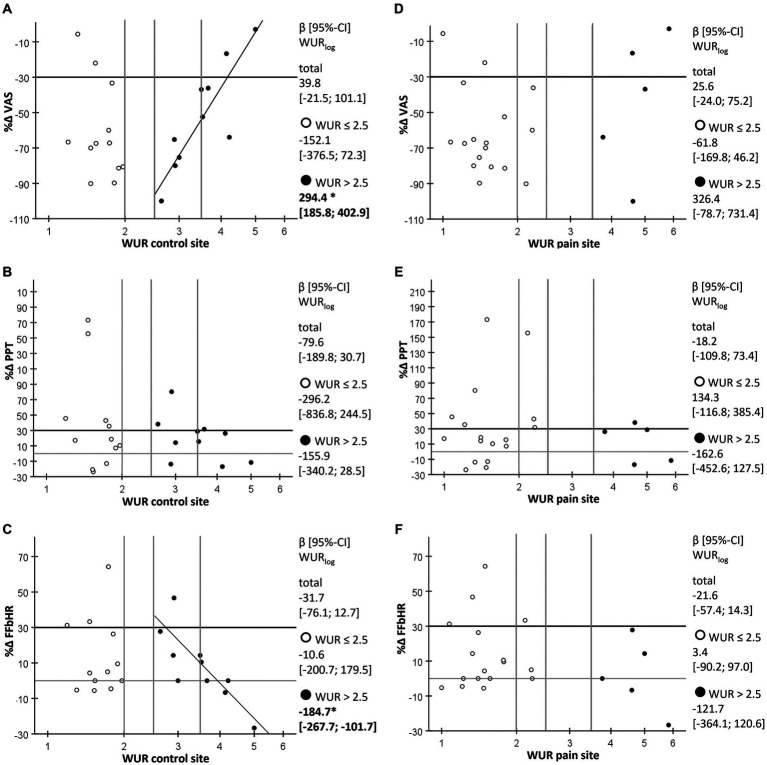
Scatterplot of percent change in pain intensity, PPT and FFbHR-scores after ten acupuncture treatments against TS at the control and the pain site. **(A)** Percent change in current pain intensity (%∆ VAS) as evaluated by the visual analog scale against the wind-up ratio (WUR) as a measure for temporal summation of pain (TS) at the control site with high TS (WUR > 2.5) indicating central sensitization. **(B)** Percent change in pressure pain threshold (%∆ PPT) against the WUR at the control site. **(C)** Percent change in the sum score of the Hannover functional ability questionnaire (%∆ FFbHR) against the WUR at the control site. **(D)** Percent change in current pain intensity (%∆ VAS) as evaluated by the visual analog scale against the WUR at the pain site. **(E)** Percent change in pressure pain threshold (%∆ PPT) against the WUR at the pain site. **(F)** Percent change in the sum score of the Hannover functional ability questionnaire (%∆ FFbHR) against the WUR at the pain site; empty circles, cases with WUR ≤ 2.5; filled circles, cases with WUR > 2.5; β [95%-CI] WURlog, regression coefficient of logarithmized WUR as estimated by generalized linear model; ^*^, significant with *p*-value < 0.001 (other associations were not significant with *p*-values > 0.05).

The ITT-data revealed linear associations between TS a the control site and the reduction of the pain intensity (β [95%-CI] WUR_log_ 294.4 [185.8; 402.9], *p* < 0.001) as well as with the improvement in physical functioning (β [95%-CI] WUR_log_ − 184.7 [−267.7; −101.7], *p* < 0.000) after ten acupuncture treatments only within the subgroup characterized by a TS at the control site of WUR > 2.5 (*n* = 10). In contrast, within patients with a WUR ≤ 2.0 (*n* = 12), response to a series of acupuncture treatments and TS was not associated. There were also no linear relationships between TS at the pain site and any of the outcomes, neither in the total sample nor in the two strata with low and high TS (Figure 4).

There were no important differences in patient characteristics and baseline values of outcomes between patients with a reduction in pain intensity of at least 30% or 50% and those without such response after a series of ten acupuncture treatments (Supplementary Table 3).

## 4 Discussion

The results presented here suggest, that the previously observed prediction of a clinically relevant immediate acupuncture response in young and middle aged chronic pain patients by an elevated TS (23) cannot be transferred to chronic LBP patients. Our primary hypothesis was not confirmed. We had hypothesized, that chronic LBP patients aged 50 years or younger with a high TS (WUR > 2.5) at a pain-free control site (dorsum of the dominant hand) would be more likely to show a clinically relevant reduction in pain intensity (≥30%) immediately after a single acupuncture treatment than patients with a low TS. Responder rates and overall immediate pain reduction was similar among chronic LBP with high and low TS at the pain free control site. Furthermore, high TS at the pain site at the lower back area was not significantly associated with the immediate response to acupuncture. The percentage of patients with a higher immediate reduction in pain intensity was descriptively even higher among those with low TS at the pain site. A single acupuncture treatment did not affect the PPT at the pain site irrespective of TS at the control and the pain site.

Study part two did not reveal a consistent association between TS at the pain free control site and any of the outcome measures. There was a close negative association between TS and the reduction in pain intensity and the improvement in physical functioning among patients with a WUR > 2.5 at the control site. However, this finding pertains to ten cases only of which four were included unintendedly due to a protocol violation and should thus not be overstated. Still venturing an interpretation, one might hypothesize that a polynomial or spline function would best describe the relationship between the response to a series of acupuncture treatments and TS (with a minimum around WUR 2.5). A recent study by another research group did not find a linear relationship between heat induced TS at the thenar eminence and the reduction in pain intensity after a series of acupuncture treatments in LBP patients (51). The authors however did not explore non-linear associations and did not provide an informative graph of their data. In line with study part one, a low TS at the pain site also tended to be associated with the response to a series of ten acupuncture treatments.

Overall, patients in this study responded well to acupuncture with a median absolute reduction of 17.5 VAS-points of the current pain intensity after one treatment (47.2% median percentage change) and a median absolute reduction of 35.5 VAS-points of last week’s pain after ten treatments (65.9% median percentage change). This effect is in the upper range of acupuncture effects on non-specific LBP-pain observed in large clinical trials (52–54). Furthermore, the proportion of patients with normal physical functioning increased from 53 to 82% after ten acupuncture treatments. The PPT at the pain site did not yet change after one acupuncture treatment, but was significantly elevated by 0.7 kg/cm^2^ (17.9% median percent change) after a series of ten acupuncture treatments. This is still in the lower range of effects on the PPT that have been observed in studies on body acupuncture or dry needling (41, 42, 55), but for example Leite et al. did not observe any change of the PPT after ten electroacupuncture treatments with a similar point regimen as in our study (56). Additionally, post treatment PPT of 4 kg/cm^2^ was still markedly below the PPT at paravertebral measure sites at the lumbar spine in healthy persons [around 5 kg/cm^2^ (35)].

The main difference between the results of our previous and our present study is a much higher responder rate (≥30% immediate pain reduction after one treatment) among patients with a low TS. Responder rates were substantially larger among patients with a low TS at the control site in the present study than among similarly aged patients with low TS at the control site in the previous study (72% vs. 18%). Conversely, responder rates among patients with a high TS at the control site were similar in the present and in the previous study (58% vs. 50%). The same accounts for the comparison of responder rates between the two studies in patients with a low TS at the pain site (73% vs. 23%) and high TS at the pain site (47% vs. 41%). This implies that treatment of chronic non-specific LBP patients with acupuncture can be recommended irrespective of their TS.

The relationship between TS and the response to acupuncture may differ between pain conditions. Future research needs to explore in which chronic pain conditions TS may predict the response to acupuncture; e.g., chronic widespread pain vs. localized pain conditions or nociceptive vs. nociplastic vs. neuropathic pain. Patients in the present study suffered exclusively from non-specific LBP, while the mixed population of chronic pain patients in the previous study included also cases with multiple pain sites and diagnoses as well as specific pain conditions. Specific causes of pain (trauma, degeneration, inflammation or nerve damage) and stage of chronicity may affect the relationship between TS and the acupuncture response. The diverging results of the present and our previous study can be discussed in the light of further patient and treatment specific aspects.

First, one might argue that patients in the present study were less advanced in their pain disorder which could have contributed to their overall high acupuncture response. Patients in the previous study were all taking part in a multimodal pain program in a university outpatient pain clinic (tertiary care), and over half of the patients showed a high degree of chronicity. In the present study, only 5% of patients showed clinically relevant impairments in physical functioning as evaluated by the FFbHR despite similar pain intensities as the population in our previous study (median [IQR] on VAS/VRS (0–100) of current pain 45 [33; 55] vs. 45 [30; 60] and average pain 59 [50.0; 63] vs. 50 [40; 60]). Thus, patients in the present study might have had good pain coping abilities, which has been identified to be at least positively associated with the response to an acupuncture series in LBP patients (51, 57). Furthermore, it appears possible that a more functional endogenous pain control, whose activation is one of the primary mechanisms of acupuncture analgesia, could have contributed to a better immediate acupuncture response among patients of the present study. Therefore, investigating the relationship between CPM and immediate acupuncture effects on pain seems of particular interest. One recent trial in LBP patients showed no linear relationship between the CPM and the change in pain of chronic LBP patients after a series of acupuncture treatments (51). However, restauration of impaired CPM (58) over a series of acupuncture treatments might decouple the baseline CPM from effects of an acupuncture series despite its potential relationship with the immediate acupuncture effects.

Second, the more intense acupuncture regimen in the present study (13 to 22 needles) might have been more optimal for chronic LBP patients without prominent sensitization (normal/low TS) than the rather reticent needling regimen in the previous study (five to ten needles). This might explain in particular the high response rates in this subgroup. More needles seem to be associated with larger effect sizes (59). At the same time the treatment did not seem to be overly intense for highly sensitized patients. Other researchers had proposed that intensity of acupuncture treatments should be adapted according to their level of sensitization, as they had observed an association between poor acupuncture responses and low electrical and pressure pain thresholds in fibromyalgia and scar pain patients (60, 61). This cannot be conjectured for the population of chronic non-specific LBP patients aged 50 years or younger in our study. Responder rates were high irrespective of TS and also irrespective of the PPT. An additional explanation for this result could be that non-specific LBP can generally be targeted well by the semi-standardized acupuncture regimen applied here. It was designed during the planning of the large health insurance sponsored German acupuncture trials by several renown acupuncture experts. The combination of local and distal points chosen according to TCM theory and segmental organization might indeed be best suited for the homogeneous patient population included in our study. In other less locally circumscribed or more complex pain conditions, that characterized the population of our previous study, a more individual acupuncture approach might be needed. Such approach might even be developed during the course of a treatment series which might explain, why effects of the first acupuncture treatment varied more in our previous study.

The external validity of our results extends only to patients with non-specific chronic LBP aged 50 years or younger without other pain conditions and general good physical function. The acupuncture regimen applied here followed the protocol of the large German acupuncture trials (43) which had been designed also in consultation with the senior author of this article. This semi-standardized regimen combines distant acupuncture points (at non-pain-sites), segmental and local acupuncture points as well as facultative points chosen according to the patients’ constitution and current situation. The overall beneficial treatment effects observed in our study confirm its suitability. We neither identified TS nor any other patient characteristic to be predictive for the overall good acupuncture response. Thus, our results do not suggest any restrictions to the application of acupuncture to such patients in routine care. Previous studies also do not give rise to the presumption that acupuncture response in chronic LBP would be associated with the patients’ age (9, 51) or pain duration (9, 11). The role of potential interactions between the use of analgesics and acupuncture treatments might require further exploration. We are only aware of one study that identified lower responses to acupuncture in chronic LBP patients using narcotics (without further specification) (9) but not in those using other medications. Comparison to our results is limited as patients in our study only used non-opioid analgesics.

### 4.1 Limitations

Despite the fact that this study clearly answers its primary research question, there are some limitations to be discussed. First, the proportion of patients with a pain reduction of at least 30%, both immediately after one and also after ten acupuncture treatments, was extremely high, and the proportion of patients with an elevation of the PPT and the FFbHR score of at least 30% was very low. This lowered the power of our responder analysis. Nevertheless, our results clearly contrast our primary hypothesis that high TS at the control site would be associated with a higher chance for a clinically relevant acupuncture response. The respective OR were below one for all outcomes. Therefore, our conclusion that the finding of our previous study cannot be generalized is fully supported by the results of the present study. Second, unmeasured confounders could have impacted our results. However, the most important confounders suggested in the scientific literature [age, sex, pain duration, baseline pain, baseline PPT and use of analgesics (9, 10)] were addressed. We did not explore the impact of expectation on our results. However, previous studies suggest that patient expectations do not seem to explain an important part of the variance in acupuncture effects on LBP (7, 9, 62–64). Furthermore, risk factors for non-specific LBP such as lifestyle, obesity, physical workload and depressive mood were not taken into account (65–69). Their potential impact on the acupuncture treatment response remains unexplored.

### 4.2 Implications for future research

As many factors, especially the risk factors for pain chronification just mentioned, can affect peripheral nerve fiber function and drive central sensitization, they should be considered in future research addressing sensory signs as predictors for the acupuncture treatment response. Generally, the discrepancies between our two studies and the fact that also previous research did not identify eminent predictors for the acupuncture response suggest a rather complex prediction network. When continuing this research, it appears advisable to promote large multicenter trials that allow a standardized collection of multifaceted data in much larger and diverse patient populations. Only such data would allow for the development of multifactor prediction models that are needed as a basis for informed treatment decisions.

The importance of approaches to precision medicine cannot be overrated. Applying ineffective treatments contributes to the risk of further pain chronification not least due to the deterioration of the patients’ confidence in a possible improvement. Furthermore, despite the fact that acupuncture can be considered fairly safe, it still bears the risk also for significant adverse events (70). For proper risk benefit considerations, more precise estimations of the expected benefits through acupuncture are crucial. In addition, human and financial resources need to be allocated efficiently in order to provide the best possible care to a maximum number of patients. Given the high prevalence of chronic pain, especially low-back pain, it is a moral obligation of the research community to continue building the path toward an individualized treatment.

## 5 Conclusion

Our results do not suggest an important role of TS for predicting a clinically important acupuncture effect or the response to a series of ten acupuncture treatments in patients with chronic non-specific LBP. Overall high response rates imply that acupuncture is a suitable treatment option for LBP patients irrespective of their TS.

## Data Availability

The raw data supporting the conclusions of this article will be made available by the authors, without undue reservation.
