# Supplementary material for: Temporal summation does not predict the acupuncture response in patients with chronic non-specific low back pain
Source: Front Neurol. 2024 Aug 23;15:1335356. doi: 10.3389/fneur.2024.1335356 (PMC11378649; doi:10.3389/fneur.2024.1335356)
Supplement: Supplementary file 1 [file Table_1.docx]

Supplementary Tables

**Temporal Summation does Not Predict the Acupuncture Response in Patients with Chronic Non-specific Low Back Pain**

Petra Bäumler, Margherita Schäfer, Luise Möhring, Dominik Irnich

## Supplementary table 1: Acupuncture points needled in the 1st and 2nd to 10th treatment

|  | 1^st^ acupuncture treatment (60 patients) | | | | 2^nd^ to 10^th^ acupuncture treamtent  (213 treatments in 25 patients) | | | |
| --- | --- | --- | --- | --- | --- | --- | --- | --- |
|  | right | left | bilateral | total | right | left | bilateral | total |
| BL 23 | - | - | 54 (90) | 54 (90) | - | - | 167 (78) | 167 (78) |
| BL 25 | - | - | 54 (90) | 54 (90) | - | - | 200 (94) | 200 (94) |
| BL40 | 2 (3) | - | 44 (73) | 46 (77) | 2 (1) | - | 161 (76) | 163 (77) |
| GB 34 | 6 (10) | 3 (5) | 26 (43) | 35 (58) | 17 (8) | 15 (7) | 63 (30) | 95 (45) |
| KI 3 | 1 (2) | 2 (3) | 29 (48) | 32 (53) | 13 (6) | 5 (2) | 109 (51) | 127 (60) |
| BL 27 | - | - | 28 (47) | 28 (47) | - | 1 (0.5) | 100 (47) | 101 (47) |
| LR 3 | 4 (7) | 6 (10) | 16 (27) | 26 (43) | 14 (7) | 13 (6) | 67 (31) | 94 (44) |
| GV 4 | 0 (43) | - | - | 26 (43) | - | - | - | 88 (41) |
| BL 26 | - | - | 22 (37) | 22 (37) | - | - | 87 (41) | 87 (41) |
| BL 24 | - | - | 22 (37) | 22 (37) | - | - | 56 (26) | 56 (26) |
| SI 3 | 4 (7) | 3 (5) | 9 (15) | 16 (27) | 14 (7) | 5 (2) | 34 (16) | 53 (25) |
| GV 20 | 0 (27) | - | - | 16 (27) | - | - | - | 40 (23) |
| BL 60 | 3 (5) | 3 (5) | 10 (17) | 16 (27) | 9 (4) | 13 (6) | 47 (22) | 69 (32) |
| BL 28 | - | - | 13 (22) | 13 (22) | - | 1 (0.5) | 64 (30) | 65 (31) |
| GV 14 | 0 (20) | - | - | 12 (20) | - | - | - | 24 (11) |
| GB 31 | 1 (2) | - | 7 (12) | 8 (13) | 1 (0.5) | - | 12 (6) | 13 (6) |
| BL 52 | - | - | 8 (13) | 8 (13) | - | - | 32 (15) | 32 (15) |
| BL 22 | 1 (2) | - | 7 (12) | 8 (13) | - | - | 19 (9) | 19 (9) |
| BL 20 | - | - | 8 (13) | 8 (13) | 2 (1) | - | 22 (10) | 24 (11) |
| GB 30 | 2 (3) | - | 4 (7) | 6 (10) | - | 1 (0.5) | 12 (6) | 13 (6) |
| BL 32 | - | - | 6 (10) | 6 (10) | - | - | 28 (13) | 28 (13) |
| BL 30 | 2 (3) | - | 4 (7) | 6 (10) | - | - | 16 (8) | 16 (8) |
| BL 54 | - | - | 5 (8) | 5 (8) | 1 (0.5) | - | 17 (8) | 18 (8) |
| BL 29 | - | - | 5 (8) | 5 (8) | - | - | 15 (7) | 15 (7) |
| BL 21 | - | - | 5 (8) | 5 (8) | 2 (1) | 2 (1) | 21 (10) | 25 (12) |
| BL 62 | - | - | 4 (7) | 4 (7) | 1 (0.5) | 3 (1) | 9 (4) | 13 (6) |
| GV 3 | 0 (2) | - | - | 1 (2) | - | - | - | 7 (3) |
| BL 53 | 1 (2) | - | - | 1 (2) | - | - | 4 (2) | 4 (2) |
| BL 34 | - | - | 1 (2) | 1 (2) | - | - | - | - |
| Ex-B 2 | - | - | 1 (2) | 1 (2) | - | - | - | - |
| KI 7 | - | - | - | - | - | - | 1 (0.5) | 1 (0.5) |
| GB 41 | - | - | - | - | - | - | 1 (0.5) | 1 (0.5) |
| Ex-B 9 | - | - | - | - | - | - | - | - |
| GV 6 | - | - | - | - | - | - | - | 1 (0.5) |
| GV 5 | - | - | - | - | - | - | - | - |
| BL 51 | - | - | - | - | 2 (1) | - | 3 (1) | 5 (2) |
| BL 50 | - | - | - | - | 2 (1) | - | 2 (1) | 4 (2) |
| BL 33 | - | - | - | - | - | - | - | - |
| BL 31 | - | - | - | - | - | - | 5 (2) | 5 (2) |
| ear zone lumbar spine | 20 (33) | 18 (30) | - | 38 (63) | 81 (38) | 49 (23) | - | 130 (61) |
| ear (55) shenmen | 17 (28) | 13 (22) | - | 30 (50) | 79 (37) | 40 (19) | - | 119 (56) |
| ear (29b) Jerome | 9 (15) | 12 (20) | - | 21 (35) | 36 (17) | 21 (10) | - | 57 (27) |
| ear vegetative rim lumbar spine | 10 (17) | 7 (12) | - | 17 (28) | 40 (19) | 32 (15) | - | 72 (34) |
| ear (56) pelvis | 4 (7) | 5 (8) | - | 9 (15) | 15 (7) | 14 (7) | - | 29 (14) |
| GB 21 | 2 (3) | 2 (3) | 4 (7) | 8 (13) | 1 (0.5) | 2 (1) | 16 (8) | 19 (9) |
| GB 39 | - | - | 6 (10) | 6 (10) | 2 (1) | - | 31 (15) | 33 (15) |
| GV 2 | 0 (8) | - | - | 5 (8) | - | - | - | 22 (10) |
| Ex-UE 7 | 1 (2) | 4 (7) | - | 5 (8) | 2 (1) | 1 (0.5) | 2 (0) | 5 (2) |
| ST 36 | 3 (5) | 1 (2) | 1 (2) | 5 (8) | 7 (3) | 10 (5) | 2 (1) | 19 (9) |
| SP 6 | - | - | 5 (8) | 5 (8) | 4 (2) | 9 (4) | 22 (10) | 35 (16) |
| BL18 | - | 1 (2) | 3 (5) | 4 (7) | 1 (0.5) | - | 21 (10) | 22 (10) |
| Ashi sacroiliac joint | 2 (3) | 1 (2) | - | 3 (5) | 1 (0.5) | - | - | 1 (0.5) |
| BL 58 | - | - | 3 (5) | 3 (5) | - | - | 4 (2) | 4 (2) |
| ear (52) sciatia | 1 (2) | 2 (3) | - | 3 (5) | 5 (2) | 8 (4) | - | 13 (6) |
| ear zone lumbar spine 2nd distal needle | 2 (3) | 1 (2) | - | 3 (5) | 6 (3) | 6 (3) | - | 12 (6) |
| ear (29) occiput point | 3 (5) | - | - | 3 (5) | 22 (10) | 8 (4) | - | 30 (14) |
| Ashi coccyx | 1 (2) | 1 (2) | - | 2 (3) | 1 (0.5) | - | - | 1 (0.5) |
| BL 57 | - | - | 2 (3) | 2 (3) | 2 (1) | 1 (0.5) | 9 (4) | 12 (6) |
| LI 11 | - | - | 2 (3) | 2 (3) | - | 1 (0.5) | - | 1 (0.5) |
| LI 4 | - | 1 (2) | 1 (2) | 2 (3) | 3 (1) | - | 2 (1) | 5 (2) |
| ear (56) sacroiliac joint 2nd needle | 1 (2) | 1 (2) | - | 2 (3) | 1 (0.5) | 2 (1) | - | 3 (1) |
| EX-NP 67 | - | - | 2 (3) | 2 (3) | 1 (0.5) | 3 (1) | - | 4 (2) |
| Yamamoto D zone | 1 (2) | 1 (2) | - | 2 (3) | 2 (1) | 4 (2) | - | 6 (3) |
| Ex-UE 8 | - | 1 (2) | 1 (2) | 2 (3) | - | 2 (1) | 2 (1) | 4 (2) |
| TE 3 | 1 (2) | - | - | 1 (2) | - | - | 1 (0.5) | 1 (0.5) |
| BL 10 | 1 (2) | - | - | 1 (2) | - | - | 1 (0.5) | 1 (0.5) |
| BL 11 | - | - | 1 (2) | 1 (2) | - | - | - | - |
| BL 19 | - | 1 (2) | - | 1 (2) | 2 (1) | - | 2 (1) | 4 (2) |
| BL 36 | - | - | 1 (2) | 1 (2) | 3 (1) | 3 (1) | 6 (3) | 12 (6) |
| BL 37 | - | - | 1 (2) | 1 (2) | 1 (0.5) | - | 10 (5) | 11 (5) |
| LR 15 | 1 (2) | - | - | 1 (2) | - | - | - | - |
| GV 8 | 0 (2) | - | - | 1 (2) | - | - | - | 6 (3) |
| ear (13) ACTH | - | 1 (2) | - | 1 (2) | 2 (1) | 1 (0.5) | - | 3 (1) |
| ear zone cervical spine | - | 1 (2) | - | 1 (2) | 5 (2) | 4 (2) | - | 9 (4) |
| ear (91) colon | 1 (2) | - | - | 1 (2) | 2 (1) | 1 (0.5) | - | 3 (1) |
| ear zone sacral spine | 1 (2) | - | - | 1 (2) | 1 (0.5) | 2 (1) | - | 3 (1) |
| ear (35) sun | 1 (2) | - | - | 1 (2) | - | - | - | - |
| ear vegetative rim cervical spine | - | 1 (2) | - | 1 (2) | 4 (2) | 4 (2) | - | 8 (4) |
| GB 20 | - | 1 (2) | - | 1 (2) | 2 (1) | 3 (1) | 3 (1) | 8 (4) |
| GB 36 | 1 (2) | - | - | 1 (2) | - | - | 1 (0.5) | 1 (0.5) |
| HT 3 | - | - | 1 (2) | 1 (2) | - | - | - | - |
| HT 7 | - | - | 1 (2) | 1 (2) | 1 (0.5) | - | 2 (1) | 3 (1) |
| LIV 5 | 1 (2) | - | - | 1 (2) | - | - | - | - |
| SI 6 | - | - | 1 (2) | 1 (2) | - | - | - | - |
| SI 9 | - | - | 1 (2) | 1 (2) | 1 (0.5) | - | 3 (1) | 4 (2) |
| ear zone thoracic spine | - | - | - | - | 5 (2) | 3 (1) | - | 8 (4) |
| ST 40 | - | - | - | - | 1 (0.5) | 5 (2) | - | 6 (3) |
| SP 9 | - | - | - | - | 2 (1) | 3 (1) | 1 (0.5) | 6 (3) |
| GB 29 | - | - | - | - | 2 (1) | 1 (0.5) | 2 (1) | 5 (2) |
| dorsal ear zone lumbar spine | - | - | - | - | 3 (1) | 1 (0.5) | - | 4 (2) |
| ear (64) shoulder | - | - | - | - | 1 (0.5) | 2 (1) | - | 3 (1) |
| Ex-B 8 | - | - | - | - | - | - | - | 3 (1) |
| Ashi (undefined) | - | - | - | - | 1 (0.5) | - | 1 (0.5) | 2 (1) |
| BL 17 | - | - | - | - | - | - | 2 (1) | 2 (1) |
| BL 44 | - | - | - | - | - | - | 2 (1) | 2 (1) |
| BL 47 | - | - | - | - | - | - | 2 (1) | 2 (1) |
| GV 9 | - | - | - | - | - | - | - | 2 (1) |
| ear (57) hip | - | - | - | - | 1 (0.5) | 1 (0.5) | - | 2 (1) |
| ear psychotropic point 2 | - | - | - | - | 2 (1) | - | - | 2 (1) |
| ear psychotropic point 3 | - | - | - | - | 1 (0.5) | 1 (0.5) | - | 2 (1) |
| ear ganglion stellatum | - | - | - | - | 1 (0.5) | 1 (0.5) | - | 2 (1) |
| ear (26a) thalamus | - | - | - | - | 1 (0.5) | 1 (0.5) | - | 2 (1) |
| KI 5 | - | - | - | - | 1 (0.5) | - | 1 (0.5) | 2 (1) |
| TE 17 | - | - | - | - | 1 (0.5) | - | - | 1 (0.5) |
| TE 5 | - | - | - | - | - | - | 1 (0.5) | 1 (0.5) |
| Ashi lumbar spine | - | - | - | - | - | 1 (0.5) | - | 1 (0.5) |
| BL 13 | - | - | - | - | 1 (0.5) | - | - | 1 (0.5) |
| BL 15 | - | - | - | - | - | 1 (0.5) | - | 1 (0.5) |
| BL 16 | - | - | - | - | - | 1 (0.5) | - | 1 (0.5) |
| BL 59 | - | - | - | - | - | - | 1 (0.5) | 1 (0.5) |
| BL 65 | - | - | - | - | - | - | 1 (0.5) | 1 (0.5) |
| BL 66 | - | - | - | - | - | - | 1 (0.5) | 1 (0.5) |
| EX-UE 1 | - | - | - | - | - | - | - | 1 (0.5) |
| ear (82) zero point | - | - | - | - | - | 1 (0.5) | - | 1 (0.5) |
| ear analgesia point | - | - | - | - | - | 1 (0.5) | - | 1 (0.5) |
| ear (101) bronchia | - | - | - | - | 1 (0.5) | - | - | 1 (0.5) |
| ear (98) spleen | - | - | - | - | 1 (0.5) | - | - | 1 (0.5) |
| ear muscle relaxation point | - | - | - | - | 1 (0.5) | - | - | 1 (0.5) |
| ear (95) kidney | - | - | - | - | - | 1 (0.5) | - | 1 (0.5) |
| ear psychotropic point 1 | - | - | - | - | 1 (0.5) | - | - | 1 (0.5) |
| ear psychotropic point 4 | - | - | - | - | 1 (0.5) | - | - | 1 (0.5) |
| ear (58) uterus | - | - | - | - | 1 (0.5) | - | - | 1 (0.5) |
| ear vegetative rim thoracic spine | - | - | - | - | 1 (0.5) | - | - | 1 (0.5) |
| ear weather point | - | - | - | - | 1 (0.5) | - | - | 1 (0.5) |
| Ex-UE (undefined) | - | - | - | - | - | 1 (0.5) | - | 1 (0.5) |
| LU 5 | - | - | - | - | - | - | 1 (0.5) | 1 (0.5) |
| ST 38 | - | - | - | - | - | 1 (0.5) | - | 1 (0.5) |
| ST 44 | - | - | - | - | - | 1 (0.5) | - | 1 (0.5) |
| SP 4 | - | - | - | - | 1 (0.5) | - | - | 1 (0.5) |
| scar region | - | - | - | - | 1 (0.5) | - | - | 1 (0.5) |
| myofascial trigger point | - | - | - | - | 1 (0.5) | - | 1 (0.5) | 1 (0.5) |

## Supplementary table 2: Full and adjusted logistic regression models for associations between immediate response to 1st acupuncture and high TS at the control and at the pain site

| Model specification | Dependent variable: %∆ VAS ≥ 30% | | | | | | | | |
| --- | --- | --- | --- | --- | --- | --- | --- | --- | --- |
|  | Covariate | - | Sex (ref = male)  Age (a) | Pain duration (m) | Use of analgesics (ref = no) | Baseline  VAS (VAS 0 - 100) | Baseline  pain last week (VAS 0 - 100) | Baseline  PPT_log_ | Baseline FFbHR |
| WUR >2.5 CS | OR  [95%-CI]  p-value | 0.54  [0.18; 1.60]  p = 0.266 | 0.56  [0.19; 1.71]  p = 0.313 | 0.57  [0.18; 1.75]  p = 0.325 | 0.48  [0.16; 1.49]  p = 0.206 | 0.56  [0.19; 1.67]  p = 0.295 | 0.55  [0.18; 1.66]  p = 0.289 | 0.54  [0.18; 1.67]  p = 0.286 | 0.54  [0.18; 1.61]  p = 0.270 |
| Covariate | OR  [95%-CI]  p-value | - | 0.72  [0.19; 2.73]  p = 0.627  0.98  [0.93; 1.03]  p = 0.380 | 1.00  [0.99; 1.01]  p = 0.714 | 2.23  [0.65; 7.58]  p = 0.201 | 1.01  [0.98; 1.04]  p = 0.401 | 0.99  [0.94; 1.04]  p = 0.751 | 0.06  [0.00; 1.14]  p = 0.061 | 1.00  [0.96; 1.05]  p = 0.888 |
| Intercept | OR  [95%-CI]  p-value | 2.60  [1.25; 5.39]  p = 0.010 | 6.78 [1.00; 46.05]  p = 0.050 | 2.81  [1.21; 6.52]  p = 0.016 | 2.10  [0.96; 4.62]  p = 0.065 | 1.43  [0.31; 6.69]  p = 0.646 | 4.17  [0.20; 85.75]  p = 0.354 | 11.70  [1.90; 72.13]  p = 0.008 | 1.95  [0.03; 113.50]  p = 0.748 |
| Omnibus test | p-value | 0.266 | 0.488 | 0.503 | 0.226 | 0.376 | 0.512 | 0.078 | 0.533 |
| H-L test | p-value |  | 0.976 | 0.343 | 0.502 | 0.582 | 0.510 | 0.475 | 0.843 |
| Nagelkerkes R^2^ |  | 0.028 | 0.055 | 0.031 | 0.067 | 0.045 | 0.031 | 0.113 | 0.029 |
| Model specification | Dependent variable: %∆ VAS ≥ 50% | | | | | | | | |
|  | Covariate | - | Sex (ref = male)  Age (a) | Pain duration (m) | Use of analgesics (ref = no) | Baseline  VAS (VAS 0 - 100) | Baseline  pain last week (VAS 0 - 100) | Baseline  PPT_log_ | Baseline FFbHR |
| WUR >2.5 CS | OR  [95%-CI]  p-value | 0.76  [0.27; 2.13]  p = 0.598 | 0.78  [0.27; 2.28]  p = 0.656 | 0.80  [0.27; 2.32]  p = 0.676 | 0.71  [0.25; 2.05]  p = 0.531 | 0.76  [0.27; 2.16]  p = 0.612 | 0.79  [0.28; 2.25]  p = 0.658 | 0.77  [0.27; 2.18]  p = 0.625 | 0.76  [0.27; 2.13]  p = 0.596 |
| Covariate | OR  [95%-CI]  p-value | - | 0.43  [0.12; 1.50]  p = 0.186  0.98  [0.93; 1.03]  p = 0.445 | 1.00  [0.99; 1.01]  p = 0.717 | 1.62  [0.55; 4.79]  p = 0.380 | 1.00  [0.98; 1.03]  p = 0.846 | 0.99  [0.94; 1.03]  p = 0.554 | 0.41  [0.03; 5.20]  p = 0.490 | 1.00  [0.95; 1.04]  p = 0.937 |
| Intercept | OR  [95%-CI]  p-value | 1.12  [0.58; 2.15]  p = 0.739 | 3.83  [0.63; 23.36]  p = 0.145 | 1.20  [0.56; 2.60]  p = 0.636 | 0.97  [0.46; 2.01]  p = 0.926 | 0.98  [0.23; 4.19]  p = 0.982 | 2.59  [0.15; 45.31]  p = 0.515 | 1.74  [0.42; 7.26]  p = 0.445 | 1.30  [0.03; 59.49]  p = 0.892 |
| Omnibus test | p-value | 0.598 | 0.371 | 0.814 | 0.590 | 0.854 | 0.729 | 0.684 | 0.867 |
| H-L test | p-value | - | 0.872 | 0.132 | 0.951 | 0.310 | 0.631 | 0.625 | 0.377 |
| Nagelkerkes R^2^ |  | 0.006 | 0.068 | 0.009 | 0.023 | 0.007 | 0.014 | 0.017 | 0.006 |
| Model specification | Dependent variable: %∆ PPT ≥ 30% | | | | | | | | |
|  | Covariate | - | Sex (ref = male)  Age (a) | Pain duration (m) | Use of analgesics (ref = no) | Baseline  VAS (VAS 0 - 100) | Baseline  pain last week (VAS 0 - 100) | Baseline  PPT_log_ | Baseline FFbHR |
| WUR >2.5 CS | OR  [95%-CI]  p-value | 0.89  [0.19; 4.11]  p = 0.877 | 0.93  [0.20; 4.33]  p = 0.922 | 0.98  [0.20; 4.68]  p = 0.977 | 0.75  [0.15; 3.66]  p = 0.717 | 0.92  [0.20; 4.31]  p = 0.913 | 0.71  [0.14; 3.58]  p = 0.683 | 1.02  [0.21; 5.03]  p = 0.978 | 0.87  [0.19; 4.04]  p = 0.855 |
| Covariate | OR  [95%-CI]  p-value | - | 1.09  [0.19; 6.29]  p = 0.920  0.98  [0.90; 1.06]  p = 0.593 | 1.00  [0.98; 1.01]  p = 0.611 | 3.89  [0.81; 18.60]  p = 0.089 | 1.01  [0.97; 1.05]  p = 0.660 | 1.06  [0.99; 1.14]  p = 0.079 | 0.03  [0.00; 1.61]  p = 0.084 | 0.98  [0.92; 1.05]  p = 0.642 |
| Intercept | OR  [95%-CI]  p-value | 0.16  [0.06; 0.41]  p = 0.000 | 0.28  [0.02; 3.66]  p = 0.333 | 0.19  [0.06; 0.60]  p = 0.005 | 0.09  [0.03; 0.33]  p = 0.000 | 0.10  [0.01; 0.96]  p = 0.046 | 0.00  [0.00; 0.32]  p = 0.013 | 0.76  [0.12; 4.77]  p = 0.773 | 0.57  [0.00; 125.66]  p = 0.840 |
| Omnibus test | p-value | 0.876 | 0.955 | 0.854 | 0.218 | 0.896 | 0.206 | 0.190 | 0.888 |
| H-L test | p-value | - | 0.692 | 0.746 | 0.327 | 0.033 | 0.075 | 0.618 | 0.540 |
| Nagelkerkes R^2^ |  | 0.001 | 0.010 | 0.010 | 0.091 | 0.007 | 0.094 | 0.099 | 0.007 |

## Supplementary table 2 continued

| Model specification | Dependent variable: %∆ VAS ≥ 30% | | | | | | | | |
| --- | --- | --- | --- | --- | --- | --- | --- | --- | --- |
|  | Covariate | - | Sex (ref = male)  Age (a) | Pain duration (m) | Use of analgesics (ref = no) | Baseline  VAS (VAS 0 - 100) | Baseline  pain last week (VAS 0 - 100) | Baseline  PPT_log_ | Baseline FFbHR |
| WUR >2.5 PS | OR  [95%-CI]  p-value | 0.32  [0.09; 1.07]  p = 0.064 | 0.31  [0.09; 1.06]  p = 0.062 | 0.33  [0.09; 1.14]  p = 0.080 | 0.29  [0.08; 1.01]  p = 0.052 | 0.30  [0.09; 1.03]  p = 0.056 | 0.31  [0.09; 1.04]  p = 0.059 | 0.28  [0.08; 1.01]  p = 0.052 | 0.32  [0.09; 1.07]  p = 0.064 |
| Covariate | OR  [95%-CI]  p-value | - | 0.80  [0.20; 3.11]  p = 0.746  0.97  [0.92; 1.02]  p = 0.274 | 1.00  [0.99; 1.01]  p = 0.861 | 2.27  [0.65; 7.95]  p = 0.198 | 1.02  [0.99; 1.05]  p = 0.304 | 0.98  [0.93; 1.04]  p = 0.545 | 0.04  [0.00; 0.98]  p = 0.048 | 1.00  [0.96; 1.06]  p = 0.849 |
| Intercept | OR  [95%-CI]  p-value | 2.75  [1.42; 5.32]  p = 0.003 | 8.35  [1.15; 60.35]  p = 0.036 | 2.87  [1.27; 6.45]  p = 0.011 | 2.17  [1.04; 4.52]  p = 0.039 | 1.35  [0.31; 5.89]  p = 0.689 | 7.14  [0.30; 171.90]  p = 0.226 | 14.65  [2.25; 95.46]  p = 0.005 | 1.85  [0.03; 112.55]  p = 0.769 |
| Omnibus test | p-value | 0.063 | 0.176 | 0.174 | 0.073 | 0.103 | 0.148 | 0.020 | 0.174 |
| H-L test | p-value | - | 0.780 | 0.620 | 0.820 | 0.303 | 0.817 | 0.484 | 0.988 |
| Nagelkerkes R^2^ |  | 0.078 | 0.110 | 0.079 | 0.116 | 0.101 | 0.086 | 0.170 | 0.079 |
| Model specification | Dependent variable: %∆ VAS ≥ 50% | | | | | | | | |
|  | Covariate | none | Sex  Age | Pain duration | Use of analgesics (Yes) | Baseline  current pain (VAS 0 - 100) | Baseline  pain last week (VAS 0 - 100) | Baseline  PPT_log_ | Baseline FFbHR |
| WUR >2.5 PS | OR  [95%-CI]  p-value | 0.40  [0.12; 1.36]  p = 0.142 | 0.40  [0.11; 1.40]  p = 0.152 | 0.41  [0.12; 1.44]  p = 0.163 | 0.38  [0.11; 1.32]  p = 0.127 | 0.40  [0.12; 1.35]  p = 0.140 | 0.39  [0.11; 1.33]  p = 0.131 | 0.39  [0.11; 1.35]  p = 0.138 | 0.40  [0.12; 1.36]  p = 0.142 |
| Covariate | OR  [95%-CI]  p-value | - | 0.45  [0.13; 1.60]  p = 0.218  0.98  [0.93; 1.03]  p = 0.377 | 1.00  [0.99; 1.01]  p = 0.911 | (Yes)  1.67  [0.56; 5.03]  p = 0.358 | 1.00  [0.98; 1.03]  p = 0.764 | 0.98  [0.93; 1.03]  p = 0.450 | 0.37  [0.03; 4.93]  p = 0.451 | 1.00  [0.95; 1.05]  p = 0.948 |
| Intercept | OR  [95%-CI]  p-value | 1.25  [0.69; 2.25]  p = 0.457 | 4.66  [0.73; 29.80]  p = 0.104 | 1.28  [0.61; 2.67]  p = 0.508 | 1.06  [0.53; 2.10]  p = 0.874 | 1.03  [0.26; 4.11]  p = 0.966 | 3.77  [0.20; 71.00]  p = 0.375 | 2.08  [0.49; 8.86]  p = 0.324 | 1.42  [0.03; 66.36]  p = 0.858 |
| Omnibus test | p-value | 0.133 | 0.166 | 0.322 | 0.211 | 0.309 | 0.243 | 0.243 | 0.323 |
| H-L test | p-value | - | 0.752 | 0.138 | 0.866 | 0.028 | 0.532 | 0.176 | 0.632 |
| Nagelkerkes R^2^ |  | 0.049 | 0.108 | 0.049 | 0.067 | 0.051 | 0.061 | 0.061 | 0.049 |

CS: control site; PS: pain site; WUR: wind-up ratio quantifying temporal summation (TS); VAS: visual analogue scale (0 – 100); PPT: pressure pain threshold; FFbHR: Hannover functional ability questionnaire; R2: coefficient of determination; OR: odds ratio derived from logistic regression; 95%-CI: 95% confidence interval, H-L test: Hosmer Lemeshow test

## Supplementary table 3:Comparison of patient characteristics between acupuncture responders and non-responders

|  | Study part 1: response to one acupuncture treatment | | | | | |
| --- | --- | --- | --- | --- | --- | --- |
|  | %∆ VAS ≥ 30% | |  | %∆ VAS ≥ 50% | |  |
|  | No n= 20 | Yes n = 40 | p-value | No n = 30 | Yes n= 30 | p-value |
| Female  n (%) | 16 (80) | 29 (73) | 0.753 | 25 (83) | 20 (67) | 0.233 |
| Age  median [IQR] | 27.0  [23.3; 41.5] | 25.0  [21.0; 35.0] | 0.251 | 26.0  [21.8; 42.3] | 26.5  [21.0; 33.0] | 0.366 |
| Pain duration  median [IQR] | 66.0  [19.8; 106.0] | 42.0  [12.3; 81.5] | 0.145 | 60.0  [14.8; 100.0] | 42.0  [12.5; 82.5] | 0.211 |
| Analgesic medication  n (%) | 5 (25) | 16 (40) | 0.390 | 9 (30) | 12 (40) | 0.589 |
| Current pain baseline  median [IQR] | 42.0  [33.3; 49.0] | 46.5  [32.3; 62.5] | 0.246 | 45.0  [34.8; 50.8] | 46.0  [26.8; 67.0] | 0.779 |
| Pain last week baseline  median [IQR] | 58.5  [50.3; 69.5] | 58.5  [50.0; 61.8] | 0.551 | 59.0  [50.8; 66.3] | 58.0  [49.0; 61.0] | 0.329 |
| PPT baseline  median [IQR] | 3.6  [2.6; 6.0] | 3.3  [2.2; 4.1] | 0.120 | 3.0  [2.3; 5.5] | 3.6  [2.3; 4.2] | 0.723 |
| FFbHR baseline  median [IQR] | 81.3  [76.0; 87.5] | 83.3  [75.0; 91.7] | 0.800 | 83.3  [75.0; 88.5] | 83.3  [74.0; 91.7] | 0.994 |
|  | Study part 2: response to 10 acupuncture treatments | | | | | |
|  | %∆ VAS ≥ 30% | |  | %∆ VAS ≥ 50% | |  |
|  | No n = 4 | Yes n = 18 | p-value | No n = 7 | Yes n = 15 | p-value |
| Female  n (%) | 3 (75) | 15 (83) | 1.000 | 6 (86) | 12 (80) | 1.000 |
| Age  median [IQR] | 32.0  [21.5; 41.0] | 30.5  [22.8; 44.8] | 0.579 | 38.0  [20.0; 48.0] | 29.0  [23.0; 43.0] | 0.860 |
| Pain duration  median [IQR] | 53.5  [9.0; 197.0] | 48.5  [24.3; 83.5] | 0.966 | 54.0  [15.0; 92.0] | 43.0  [13.0; 109.0] | 0.888 |
| Analgesic medication  n (%) | 1 (25) | 4 (22) | 1.000 | 2 (29) | 3 (20) | 1.000 |
| Current pain baseline  median [IQR] | 44.0  [35.3; 51.3] | 44.5  [33.8; 59.3] | 0.766 | 46.0  [40.0; 66.0] | 44.0  [33.0; 55.0] | 0.459 |
| Pain last week baseline  median [IQR] | 69.5  [54.5; 71.8] | 60.0  [48.5; 62.5] | 0.125 | 68.0  [50.0; 72.0] | 60.0  [49.0; 61.0] | 0.077 |
| PPT baseline  median [IQR] | 2.8  [2.0; 3.7] | 3.6  [2.2; 5.0] | 0.349 | 3.5  [2.1; 6.1] | 3.4  [2.1; 4.2] | 0.860 |
| FFbHR baseline  median [IQR] | 68.8  [62.5; 78.1] | 83.3  [75.0; 88.5] | 0.048 | 79.2  [62.5; 87.5] | 83.3  [75.0; 87.5] | 0.722 |

PPT: pressure pain threshold; FFbHR: Hannover functional ability questionnaire; IQR: interquartile range; %∆ VAS: percent reduction in current pain intensity as evaluated by the visual analogue scale (0 – 100); group comparisons of continuous variables by Mann-Whitney-U test and of dichotomous variables by Fisher’s test
